# ZD 1839 in patients with brain metastases from non-small-cell lung cancer (NSCLC): report of four cases

**DOI:** 10.1038/sj.bjc.6601116

**Published:** 2003-07-15

**Authors:** F Cappuzzo, C Calandri, S Bartolini, L Crinò

**Affiliations:** 1 Division of Medical Oncology, Bellaria Hospital, Via Altura 3, 40139 Bologna, Italy

**Keywords:** tyrosine kinase inhibitor, ZD 1839, non-small-cell lung cancer

## Abstract

The activity of ZD 1839 on brain metastases (BM) from Non-Small-Cell Lung Cancer (NSCLC) is unknown. We report four cases of BM responding to ZD 1839 theraphy.

ZD 1839 (gefitinib, Iressa; AstraZeneca Pharmaceuticals, Wilmington, DE, USA) is an orally active, selective epidermal growth factor receptor (EGFR) tyrosine-kinase inhibitor (TKI), which demonstrated antitumour activity *in vitro* and *in vivo* (Ciardiello *et al*, 2000). In pretreated non-small-cell lung cancer (NSCLC) patients, phase I and II studies demonstrated that ZD 1839 is active and well tolerated, with a response rate of about 10–15% (Kris *et al*, 2000,^2002^; Fukuoka *et al*, 2002). The presence of brain metastases (BM) has been considered as an exclusion criterion in all trials conducted so far (Kris *et al*, 2000,^2002^; Fukuoka *et al*, 2002; Giaccone *et al*, 2002; Johnson *et al*, 2002). Therefore, the activity of ZD 1839 on BM is unknown. Since January 2001, we participated in the ZD 1839 compassionate use programme, in which pretreated fit NSCLC patients were candidates to receive ZD 1839 at 250 mg daily dose. Compassionate use programme criteria do not exclude patients with BM. Here, we report four cases of BM from NSCLC patients responding to ZD 1839 therapy.

Patients were 53, 54, 55, and 65 years old, two male and two female patients. Histology was adenocarcinoma (two cases), squamous cell carcinoma (one case) and bronchiolo-alveolar carcinoma (one case). All patients developed BM in the presence of extracranial disease. All patients had received a first-line platinum-based chemotherapy, and two patients were treated with whole-brain radiotherapy (WBRT) for BM terminated at least 3 months before starting ZD 1839, with evidence of brain disease progression when therapy with ZD 1839 was started. In the other two patients, ZD 1839 was begun for appearance of asymptomatic BM and progressive disease on the extracranial sites. After 3 months of ZD 1839 therapy, all the patients had a partial response both in the brain and in the extracranial sites (Figure 1

**Figure 1:**
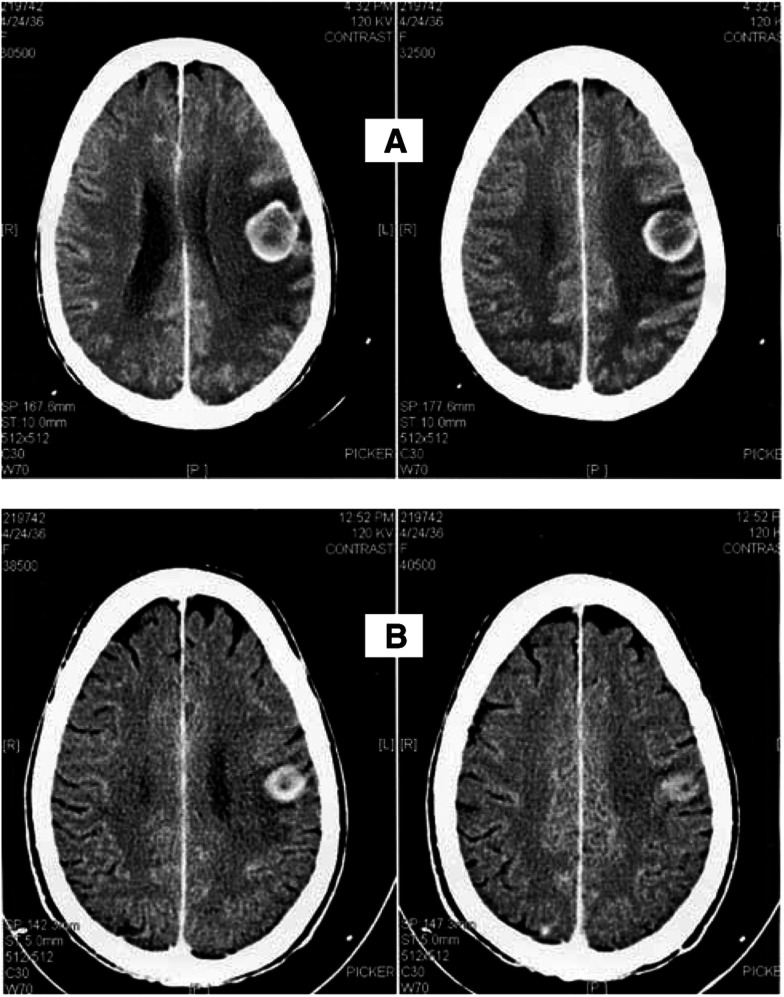
Case 4: Brain CT-scan at baseline (**A**) and after 3 months of ZD1839 therapy (**B**). Brain metastasis from NSCLC responding to ZD 1839 therapy. This patient has been pretreated with three lines of chemotherapy including platinum and taxanes, and received ZD 1839 after whole-brain radiotherapy failure.

). At the time of this analysis, two patients discontinued the treatment after 8 and 15 months for disease progression, while two patients are still on treatment with no evidence of treatment failure after 6+ and 11+ months. ZD 1839 therapy was generally well tolerated, with skin toxicity recorded in three patients (two patients grade 1 and one patient grade 2). All four patients experienced symptomatic improvement while on treatment.

This report suggests that ZD 1839 is effective in NSCLC patients with pretreated BM, but further trials are needed to better evaluate the role of this compound in these patients. Based on our results, the presence of BM should not be considered an exclusion criterion in future TKI studies.
